# Wheat historical phenotypic data from European genebanks as an important resource for research and breeding

**DOI:** 10.1038/s41597-026-06908-x

**Published:** 2026-03-02

**Authors:** Erwan Le Floch, Anne-Françoise Adam-Blondon, Michael Alaux, Etienne Bardet, Noor Bas, Filippo M. Bassi, Maja Boczkowska, Paulina Bolc, Matthijs Brouwer, Boulos Chalhoub, Reinhoud De Blok, Gergana Desheva, Jagadeeshwar R. Etukala, Raphaël Flores, Indira Galit, Wouter Groenink, Rene Hauptvogel, Roel Hoekstra, Zakaria Kehel, Paul Kersey, Renata Kowalik, Suman Kumar, Bozhidar Kyosev, Matthias Lange, Cătălin Lazăr, Cristina Marinciu, Diana Martín-Lammerding, Adrian Motor, Mounika Pachipala, Mercedes Pallero-Baena, Eugen Petcu, Aleksandra Pietrusińska-Radzio, Wiesław Podyma, Cyril Pommier, Marta Puchta-Jasińska, Szymon Puła, Laura Reiniers, Joseph Ruff, Magdalena Ruiz, Francesca Sansoni, Beate Schierscher, Gabriela Șerban, Sarah Serex, Patrizia Vaccino, Robbert Van Treuren, Mandea Vasile, Liliana Vasilescu, Andrea Visioni, Stephan Weise, Erik Wijnker, Meryem Zaim, Jochen C. Reif, Marcel O. Berkner

**Affiliations:** 1 Université Paris-Saclay, INRAE, BioinfOmics, URGI, 78026 Versailles, France; 2 Centre for Genetic Resources, the Netherlands (CGN), Wageningen University and Research, Wageningen, the Netherlands; 3 ICARDA, Biodiversity and Integrated Gene Management, P.O. Box 6299 Rabat Instituts, Rabat, Morocco; 4 Plant Breeding and Acclimatization Institute - National Research Institute, Radzików, 05-870 Błonie Poland; 5 Plant Breeding, Wageningen University & Research, P.O. Box 386, 6700 AJ Wageningen, The Netherlands; 6 Field-Crop Breeding and Genetic Resources, Agroscope, Nyon, Switzerland; 7 Agricultural Academy, Institute of Plant Genetic Resources “Konstantin Malkov”, 4122 Sadovo, Bulgaria; 8 Leibniz Institute of Plant Genetics and Crop Plant Research (IPK), Gatersleben, Germany; 9 National Agricultural Research and Development Institute Fundulea (NARDI), Breeding Cereals Department, N. Titulescu street, no. 1, Fundulea, 915200 Călărași, Romania; 10 National Agricultural and Food Center, Lužianky, Slovak Republic; 11 Royal Botanic Gardens, Kew, Richmond, UK; 12 Instituto Nacional de Investigación y Tecnología Agraria y Alimentaria, INIA, CSIC, 28805 Alcalá de Henares, Spain; 13 Cell Biology and Genetics, Department of Biomedicine and Biotechnology, University of Alcalá, Alcalá de Henares, Spain; 14 Council for Agricultural Research and Economics, Defence and Certification (CREA-DC), s.s. 11 to Torino, km 2.5, Vercelli, 13100 Italy; 15 Council for Agricultural Research and Economics, Research Centre for Cereal and Industrial Crops, s.s. 11 to Torino, km 2.5, Vercelli, 13100 Italy

**Keywords:** Plant breeding, Data processing, Natural variation in plants, Data publication and archiving

## Abstract

Plant genetic resources are considered a treasure trove of valuable, untapped diversity that holds the key to breeding the crops of the future. However, the use of these resources in breeding is often limited due to the lack of comprehensive phenotypic characterization. The present study provides extensive historical phenotypic data from nine genebanks as a MIAPPE compliant data set. We compiled and curated phenotypic data from 43,293 wheat accessions, encompassing 460,399 data points across 52 traits, including the three core traits of plant height, heading time, and thousand kernel weight from seven decades. The exceptional quality of the presented dataset was highlighted by predominantly high heritabilities. Phenotypic data of such quantity and quality is a crucial resource for unlocking the valuable diversity of plant genetic resources for agricultural advancement.

## Background & Summary

Plant genetic resources are key to crop improvement in response to changing consumer demands, loss of resistance to pests and diseases and changing environmental conditions as for example due to climate change^1^. However, the actual use of plant genetic resources in breeding is usually limited and in stark contrast to their potential and promise^2^. Generating and/or releasing comprehensive genotypic and phenotypic data for plant genetic resources has been proposed as a first important step to promote a more systematic use of plant genetic resources in breeding and research^3^. In line with this, individual genebanks, as key custodians of plant genetic resources, have made comprehensive genotypic and phenotypic data from their plant genetic resources collections available in pioneering studies^4–6^. In addition, systematic efforts to mine for valuable diversity in important agronomic traits have been successfully implemented^7–9^.

Interestingly, the wealth of phenotypic data for plant genetic resources is due to historical data collected during their regeneration process. The phenotyping information is needed to maintain the integrity of the seed throughout the regeneration cycles^10^. As the regeneration activities are not embedded in scientific research, and accessions are mainly grown when facing low availability of seeds in stock or when germination rates fall below threshold values, the resulting data structure is non-orthogonal. This poses challenges for data analysis and curation, but solutions have been proposed and validated using comprehensive wheat datasets^6^.

Data curation is simplified and facilitated by data standardisation that has been discussed in the genebank community and agreed upon for the passport data (Multi-crop Passport Descriptors, MCPD^11^). This enabled the joint presentation of passport data across genebanks on international platforms such as Genesys^12^ or the European Search Catalogue for Plant Genetic Resources (EURISCO)^13^, and paved the way for queries for suitable accessions at a global level. The standardisation of phenotypic data has also been a widely discussed topic since the late 1970s. For example, the IPGRI/Bioversity Descriptors for Wheat have been developed, but their consistent implementation across genebanks^14^ has yet to be achieved. More recently, the Minimum Information About a Plant Phenotyping Experiment (MIAPPE)^15^ built a lightweight data exchange standard for phenotypic experiments that has been adopted in databases^16^ with simple or complex datasets.

In this study, we undertook the effort to curate historical phenotypic data from the propagation of eight European and one international genebank, making them available for breeding and research according to the FAIR principles^17^ (Findable, Accessible, Interoperable and Reusable). In total, we analysed phenotypic data from 43,293 accessions, comprising 460,399 data points for 52 traits, including the three core traits plant height, heading time, and thousand kernel weight. The predominantly high heritabilities demonstrate the excellent quality of the phenotypic data, which is a crucial key to unlocking the valuable diversity present in the plant genetic resources. This study, as the first step in creating a substantial wheat germplasm inventory, enables breeding and research to make educated selections of germplasm and thus, foster the development towards an agriculture that can still serve human needs in the future.

## Methods

### Plant material

The study includes wheat collections from the genebanks hosted at eight European and one international institutes. At the time of the data curation, the composition and characteristics of the nine wheat collections were as outlined below for each genebank, as well as for the plant material reflected in the dataset.

The wheat collection of the Italian *Consiglio per la ricerca in agricoltura e l’analisi dell’economia agraria - Cerealicoltura e Colture Industriali* (CREA-CI) genebank comprises nearly 4,500 *T. aestivum* accessions, from landraces to modern cultivars (EURISCO^18^, accessed 2025-01-30). The phenotypic data presented in this study include 1,473 *T. aestivum* accessions, mainly winter type, corresponding to 30% of the collection.

The wheat collection of the International Center for Agriculture Research in the Dry Areas (ICARDA) genebank comprises nearly 39,026 accessions within the genus *Triticum*. Of these, 48% are *T. turgidum*, 39% are *T. aestivum*, and 10% are *T. aethiopicum*. The remaining collection is primarily composed of *T. monococcum, T. urartu, T. timopheevii, T. durum*, and *T. vavilovii*. The phenotypic data presented in this study include 22,609 accessions, which corresponds to more than 58% of the entire collection.

The wheat collection of the Polish *Instytut Hodowli I Aklimatyzacji Roślin – Państwowy Instytut Badawczy* (IHAR-PIB) genebank comprises nearly 15,038 accessions within the genus *Triticum*. The collection consists mainly of *T. aestivum* (70.59%), followed by *T. durum* (16.67%), *T. spelta* (0.79%), *T. dicoccum* (0.78%), *T. turgidum* (0.51%), *T. monococcum* (0.33%), *T. aethiopicum* (0.23%), *T. timopheevii* (0.19%), *T. compactum* (0.18%), *T. polonicum* (0.17%), *T. persicum* (0.11%), and *T. macha* (0.10%). Wild species including *T. boeoticum*, *T. dicoccoides*, and *T. araraticum* together account for 0.25%. Additionally, *T. vavilovii*, T*. carthlicum*, *T. karamyschevi*i, *T. sphaerococcum*, *T. ispahanicum*, *T. turanicum*, *T. kihare*, *T. militinae*, and *T. zhukovskyi* together make up 0.24% of the collection. For 8.86% of the accessions, the species has not yet been determined or needs verification, and these accessions are identified as *T. sp*. The phenotypic data presented in this study include 7,607 accessions (2,340 spring wheat and 5,267 winter wheat), corresponding to more than 50.6% of the entire collection.

The *Triticum* collection from the Spanish genebank of *Instituto Nacional de Investigación y Tecnología Agraria y Alimentaria* (INIA-CSIC) comprises 3,330 accessions, mainly of *T. aestivum* (56%) and *T. durum* (34%). Other species included are *T. spelta* (3.6%), *T. dicoccum* (3%), *T. monococcum* (1.5%), *T. polonicum* (0.8%), *T. timopheevii* (0.2%) and *T. carthlicum* (0.2%), as well as wild species (*T. boeoticum, T. urartu and T. dicoccoides*). The phenotypic data presented in this study include 1,260 accessions of *T. aestivum* (68% of the *T. aestivum* collection), which corresponds to 38% of the entire collection.

The Institute of Plant Genetic Resources - Sadovo (IPGR-Sadovo) genebank in Bulgaria comprises 11,810 accessions within the genus *Triticum*, representing 39 plant species. *Triticum aestivum* makes up the largest proportion of the collection (76.62%). The proportion of the other species are as follows: *T. durum* - 17.17%, *T. monococcum* - 1.27%, *T. dicoccum* - 0.64%, *T. timopheevii* - 0.19%, *T. spelta* - 0.49%, *T. polonicum* - 0.10%, *T. isphanicum* - 0.03%, *T. carthlicum* – 0.02% and wild species including *T. boeoticum* - 0.63%, *T. urartu* - 0.05%, *T. dicoccoides* - 0.13%, and *T. araraticum* - 0.02%. The phenotypic data presented in this study includes 504 winter wheat accessions, corresponding to 4.27% of the entire collection.

The wheat collection of the National Agricultural Research and Development Institute Fundulea (NARDI), Romania, comprises nearly 1,879 accessions within the genus *Triticum*, of which 1,642 samples are from *Triticum aestivum* (87.38% landraces, obsolete, and modern varieties) and 237 samples from other wheat species. The largest share of the wheat collection is occupied by *T. aestivum subsp. aestivum*, and the other species distributed as follows: *T. aegilops* (26.16%), *T. tauschii* (0.84%), *T. araraticum* (0.84%), *T. boeticum* (0.42%), *T. dicoccoides* (0.84%), *T. dicoccum* (0.42%), *T. monococcum* (15.61%), *T. petropavlovsky* (0.42%), *T. spelta* (1.69%), *T. spaerococcum* (0.42%), *T. timopheevi* (16.88%), *T. turgidum* (8.44%), *T. urartu* (7.59%), *Elymus sp*. (5.91%), *Leymus sp*. (7.17%), and *Thinopyrum sp*. (6.33%). The phenotypic data presented in this study include 750 winter wheat accessions (45.67% of total *Triticum aestivum* samples), which corresponds to 39.91% of the entire collection.

The wheat collection of the *Narodne Polnohospodarske a Potravinarske Centrum* (NPPC) genebank in Slovak Republic comprises 6,975 accessions within the genus *Triticum*. The collection is predominantly composed of *T. aestivum* (90.51%). Other species include *T. durum* (4.20%), *T. spelta* (0.92%), *T. dicoccum* (0.14%), *T. monococcum* (0.54%), *T. polonicum* (0.23%), *T. timopheevii* (0.14%) and *T. carthlicum* (0.10%). Wild species, including *T. boeoticum*, *T. urartu*, *T. dicoccoides*, and *T. araraticum*, together account for 0.43%. The phenotypic data presented in this study include 5,784 accessions (891 spring wheat and 4,893 winter wheat), which corresponds to more than 82% of the entire collection.

*Eidgenössisches Department für Wirtschaft, Bildung und Forschung* - Agroscope (WBF-Agroscope) began collecting wheat landraces and varieties from Switzerland and abroad as early as 1900. Today, the genebank holds a total of 9,241 accessions, with 70% being *T. aestivum*, 25% *Triticum spelta*, 3% *T. durum*, and few others, including *T. monococcum*, *T. dicoccum*, *T. timopheevii*, *T. spelta, T. polonicum, T. isphanicum, T. carthlicum, T. macha,* and wild species such as *T. boeoticum, T. urartu, T. dicoccoides*, and *T. araraticum*. The phenotypic data presented in this study included 495 wheat accessions, 1 *T. pyramiadale*, 2 *T. isphanicum*, and *2 T. durum* (47 spring wheat, 449 winter wheat, and 6 unclassified accessions), corresponding to more than 7% of the wheat collection. Additionally, 500 *T. spelta* accessions were phenotyped in this study, representing more than 22% of the spelt collection (2,301 accessions).

The collection of Wageningen University and Research - Centre for Genetic Resources, the Netherlands (WUR-CGN) holds 4,730 accessions belonging to the genus *Triticum*. *T. aestivum* represents the largest portion, accounting for 85% of the collection. A further 5% are *T. dicoccoides*, 3% are *T. durum*, and the remaining 7% are accessions spread across 10 other *Triticum* species. Of all *Triticum* accessions in the collection, 45% are landraces. The phenotypic data presented in this study include data of 2,239 accessions, which corresponds to 47% of the total number of accessions.

A summary of Triticum species proportions and their hosting genebanks is available in Supplementary Table 1.

### Phenotyping protocol

The primary role of genebanks is to preserve plant genetic resources and to make such genetic material available, which necessitates regular regeneration and multiplication of wheat in field plots. Seed multiplication is required when seed stocks become insufficient, germination rates fall below a critical threshold, large quantities of seed are needed for research, or when new accessions are added to the genebank.

During the regeneration process, morphological and agronomic traits are evaluated for identity purposes and potential changes between generation cycles, following strict quality guidelines. In case of uncertainty about possible morphological changes during regeneration, a comparison is usually made with the original material. This can be done, for example, using the original descriptions or by comparison with herbarium or spike specimens.

**Fig. 1:**
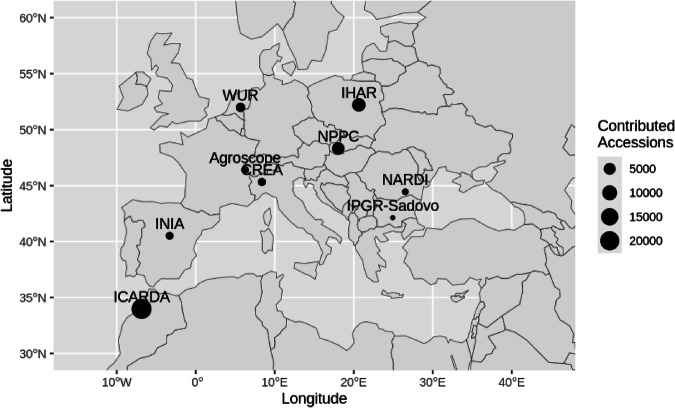
Geographic location of the nine represented genebanks with the number of contributed accessions displayed by dot size.

**Fig. 2:**
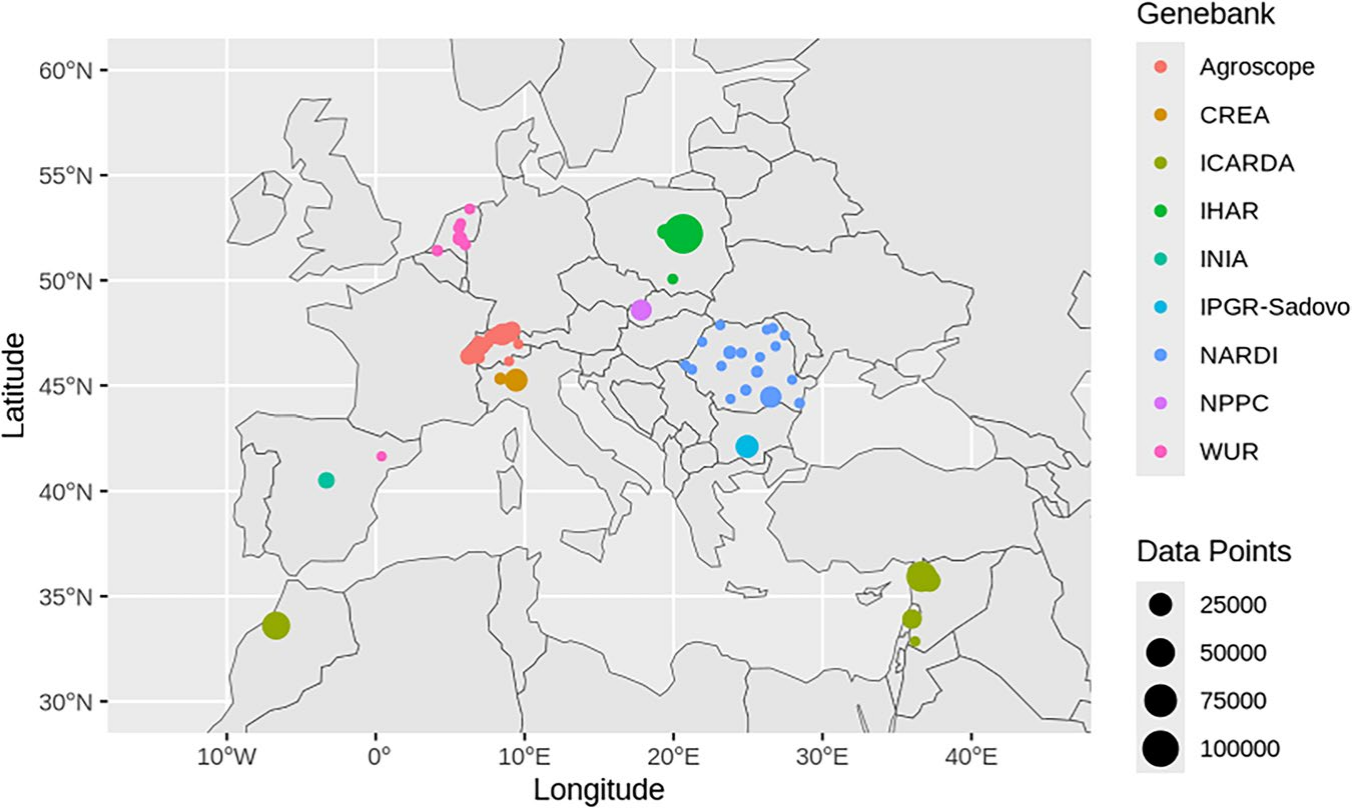
Geographic location where accession data were collected by the nine genebanks. Close locations may show overlapping data points.

We compiled data from nine genebanks (Fig. 1), with the earliest phenotypic data collected in 1967 and most recent data from 2022, gathered at various sites across Europe and North Africa (Fig. 2).

Not all accessions were regenerated each year, resulting in a non-orthogonal data structure. The experimental design can be interpreted as a randomised design with sparse replication. For seed multiplication, winter wheat accessions were usually sown from September to November, while spring wheat accessions were sown in temperate climates from February to April. In Mediterranean wheat locations, however, both spring and winter accessions were sown in winter, mainly in November.

Across all genebanks, data were recorded for heading date (HD), plant height (PH), and thousand kernel weight (TKW).

HD was assessed for wheat accessions with vernalization requirement as the number of days from 1^st^ January until when 50% of the plants reached heading (BBCH 59). At the INIA genebank, HD was expressed as days after sowing for both winter (with vernalization requirements) and spring accessions. At the NPPC genebank, winter wheat days to heading were recorded in days from sowing.

PH was assessed in centimetres from the soil surface to the top of the ear.

TKW was determined after seed harvest and expressed in grams based on approximately 15% grain moisture.

The Table 1 summarizes the phenotyping experiments. A summary of environments in which accessions were tested can be found in Supplementary Table 2.

**Table 1:** Summary of phenotyping experiments.

Genebank	Phenotyping campaigns	Locations	Accessions tested	Data points
Agroscope	73	51	1186	76392
CREA	13	3	1353	28704
ICARDA	28	7	22609	145296
IHAR	52	3	7607	128936
INIA	22	1	1260	6331
IPGR-Sadovo	31	2	504	27398
NARDI F.	40	18	750	23406
NPPC	40	2	5784	18362
WUR	21	10	2239	5574

### Phenotyping data flow

**Fig. 3:**
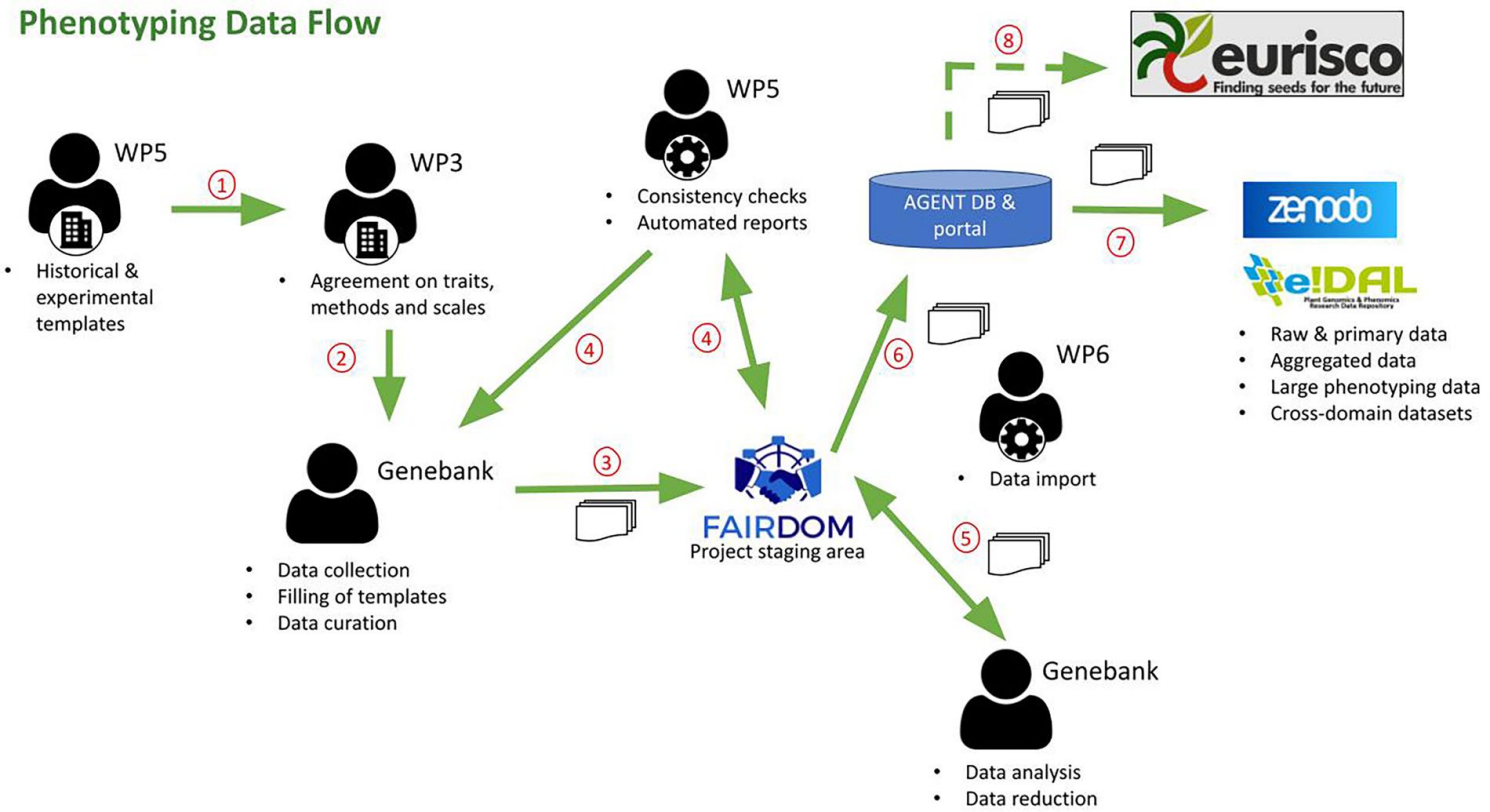
Data flow for AGENT phenotyping data. Source: AGENT guidelines for data flow^21^.

The raw phenotypic data have been curated in accordance with the *Activated GEnebank NeTwork* (AGENT)^19,20^ project guidelines for data flow^21^ (Fig. 3). Data exchange and curation used a MIAPPE^15^ compatible dedicated template^21^. The required metadata attributes were defined according to the AGENT primary data preservation and analysis scenarios, which aim to make legacy data from individual genebanks systematically available. For this purpose, characterization data on phenology, adaptation to biotic and abiotic stress, and environmental descriptors were selected, as they are sufficiently documented in genebanks and suitable for integrative analysis.

The template begins with the study, *i.e*. experiment, section which includes naming, free-text description, campaign start and end dates, and location. The observation variables section allows traits minimal description plus phenotyping protocols with the addition of valid phenotype values for some traits. When possible, cross references are made with the Crop Ontology. The plant material is identified by the triplet of FAO-WIEWS^22^ institute code, genus name and accession number, as well as the extra fields AGENT ID and, if available, Digital Object Identifier (DOI). The AGENT ID is a project internal identifier to ensure unique material identification and material provenance for sequence data submission to INSDC databases. It can be resolved using the AGENT portal^23^.

Excel templates containing both data and metadata were filled and uploaded by each genebank to a FAIRDOM^24^ instance, used as a staging area for project data. Several iterations of semi-automated metadata and data curation were made using the “Excel Validator” tool^25^, with support by dedicated bioinformatics helpdesk members, until a sufficient quality level was reached. Then the datasets underwent statistical analysis (section “Data analyses”), were imported to the AGENT database, displayed in the AGENT portal^23^ and archived in data repositories (section “Data records”).

### Data analyses

The phenotypic raw data, as curated from historical records, were analysed separately per genebank collections and furthermore, the analysis distinguished between spring and winter types based on the genebank catalogues. Data of all collections were analysed following a comparable procedure, as outlined below.

After data validation (section “Phenotyping data flow”), raw values were trimmed in order to facilitate the later analysis. The phenotypic data were filtered to exclude obvious outliers and possible recording errors, with voluntarily lax thresholds: 5 to 250 cm for PH, 50 to 250 days for HD, and 5 to 100 g for TKW. Then all phenotypic records of accessions which were tested in a single year were excluded from the dataset, as well as all years which were just represented with a single data point. In a few cases, this exclusion threshold for accessions and years was increased by one, with the aim to increase the average connectivity of the data.

The detection of outlier raw values, the estimation of heritabilities, and of best linear unbiased estimates (BLUEs) were performed in reference to the method described by Philipp *et al*.^26^. Concisely, a linear mixed model equation was fitted as follows:

1 $${y}_{{ij}}={\rm{\mu }}+{g}_{i}+{a}_{j}+{e}_{{ij}}$$

in which the phenotypic data measured on accession *i* in the year *j* is represented by the term *y*_*ij*_ which depends on the average effect of the respective genebank collection (*µ*), the effect of the accession (*g*_*i*_), and random effect of the year (*a*_*j*_). While both terms, *g*_*i*_ and *a*_*j*_, were considered random for the calculation of variance components, the term *g*_*i*_ was modelled as fixed for the outlier detection and the estimation of BLUEs. The error term, given by e_ij_, was modelled with a year-specific variance. After standardisation, the error term was used to detect outlier data points^27^; the detection included an adjustment for multiple testing^28^.

In details, the followed steps were, for each trait:

I. Extract the campaign effects, from asreml model with: fixed = trait ~campaign, random = ~accession
II. Extract heterogeneous error variances
III. Calculate and standardize the coefficient of variation
IV. Remove outlier campaigns based on the standardized coefficient of variation (threshold: 3.5)
V. Use the resulting data to make a second asreml model with fixed = trait ~ accession, random = ~ campaign
VI. Bonferroni-Holm multiple testing to mark outliers based on standardized residuals (threshold: bholm < 0.05)
VII. Remove marked outlier data points.

The extracted variance components were used to calculate heritabilities (*h*^2^) as follows:

2 $${h}^{2}=\frac{{\sigma }_{G}^{2}}{{\sigma }_{G}^{2}+\frac{{\sigma }_{e}^{2}}{{\underline{N}}_{Y}}}$$

where variance of the accession and the error variance are considered by ${\sigma }_{{\rm{G}}}^{2}$ and ${\sigma }_{{\rm{e}}}^{2}$, respectively. The average number of years with phenotypic data per accession is given by ${\bar{N}}_{Y}$.

The details of the workflow can be found in the code (see Code availability).

## Data Records

The data analysed in this study is available both as a MIAPPE compliant tabular excel format^29^ used as data analysis input (dataset version: 1.2), and as FAIR Digital Object (FDO)^30^ in the Annotated Research Context (ARC) format^31^ that can be accessed in the Plant Genomics and Phenomics Research Data Repository (e!DAL-PGP)^32^ (revision 2).

The MIAPPE tabular format includes the raw automatically and manually curated data used as input to produce the processed data. Each file gathers all the studies of a given genebank, with one file for the raw data and one for the processed data. They are self describing and include all metadata such as experiment locations, timing, accessions identification, trait definitions and methods.

The FDO was compiled with the PLANTdataHUB^33^ tools and published for collaborative improvement at the underlying GitLab instance (https://git.nfdi4plants.org/lange/Curated_wheat_historical_phenotypic_data_from_European_Genebanks). The Investigation, Study, Assay (ISA)^34^ tab-delimited (Tab) format with the technology type extension MIAPPE^15^ was used to collect and publish the complex metadata and interlink the data files. In the investigation file, information is reported on a per-column basis and the fields are organized and divided in sections. This covers general information such as contacts, protocols and equipment, and the description of used terminologies. Experiment semantics is included as well. Those are experimental factors, measure protocols, definition of parameter scales and units which are referenced in the study and assay files.

To preserve the context of historical data originated and curated by nine partner institutes, each partner data set is described in study files per year and location. Their file names prefix is the partner acronym, followed by the year and site, and are structured on a per-row basis with the first row being used for column headers. It contains contextualizing information for one or more assays, for example: the subjects studied, their source(s), the sampling methodology, their characteristics, and any treatments.

The assay files, which are linked to in the study files, are organized on a per-row basis as well and describe the measured phenotypic data. Here, all parameters and units are recorded as defined in the investigation file. The second sheet in the assay file includes outlier-corrected and BLUEs data, and finally the study comprises references to the weather data, if available.

The genotypic data with a resolution of DArTseq data, generated for 4,482 of the 43,293 accessions, have been made available online. The raw sequence data is archived at the European Nucleotide Archive (ENA^35^) at EMBL-EBI under the study accessions PRJEB49199 (main study, https://www.ebi.ac.uk/ena/browser/view/PRJEB49199) and PRJEB81686 (study of Agroscope, https://www.ebi.ac.uk/ena/browser/view/PRJEB81686).

The technical interoperability between the present and future phenotypic and genomic data is ensured by harmonized material and sample metadata^21^, as well as use of interlinked sample IDs. Here, the BioSamples ID, used to uniquely identify sequences and genotypes in variant calls, comprises the ID of used material. This material ID (called AGENT ID^21^) as well as related material IDs provided by the genebanks, like DOIs, are referred to in the BioSamples record. In a nutshell, interoperability is guaranteed by metadata compatibility and network of material, sample and dataset identifiers.

The metadata available in Recherche Data Gouv and e!DAL-PGP are findable using the WheatIS and FAIDARE data portals (https://urgi.versailles.inrae.fr/wheatis/, https://urgi.versailles.inra.fr/faidare/). It will allow the discoverability, and therefore reusability, of the data at the international level for the wheat and plant science communities, and enable data integration^37,38^.

## Technical Validation

**Fig. 4:**
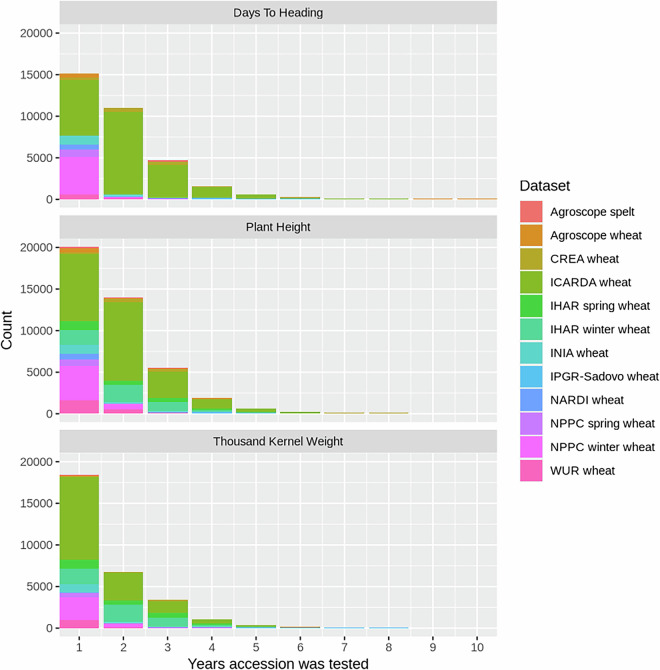
Distribution of the number of years in which accessions were regenerated. The x axis has been cropped to 10 years but a few accessions were tested as long as 36 different years, as a control trial.

**Fig. 5:**
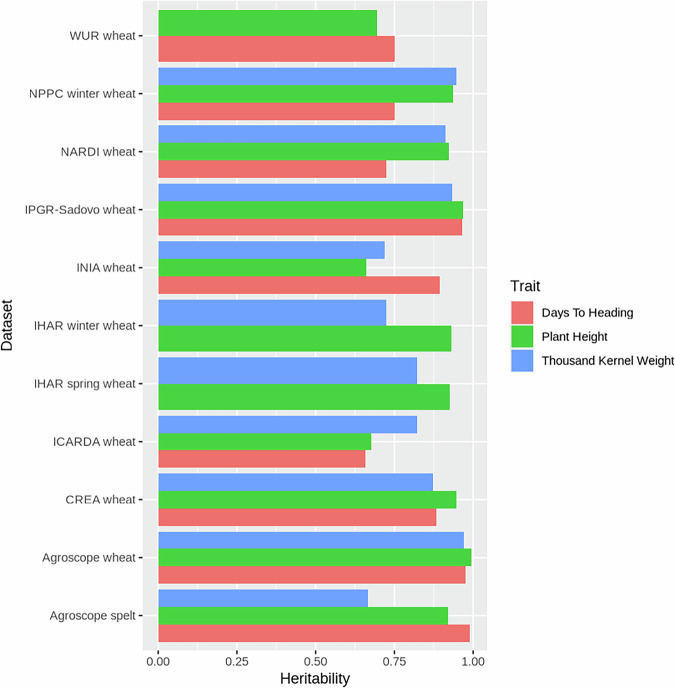
Heritability estimates of days to heading, plant height, and thousand kernel weight, for each genebank.

One challenge encountered during the analyses of the historical datasets was their non-orthogonal structure, with a substantial number of accessions tested in only one year (Fig. 4). As a central component of the validation process, we estimated the heritabilities for each trait and each genebank using a previously established methodology^6,26^. Heritability represents the proportion of the genetic variance to the phenotypic variance. In most cases, the estimated heritabilities were excellent (Fig. 5), underscoring the data quality.

The Tables 2–4 summarise for each core trait the size of the dataset, the number of accessions, the proportion of data points retained after outlier correction, and the heritabilities. In a few cases, no data points remained after initial filtering and outlier correction: specifically for the three traits in the NPPC spring wheat dataset, and thousand kernel weight in the WUR dataset. Despite this, the final curated datasets include in total 23,282 accessions maintained in the nine genebanks.

**Table 2:** **Days to heading** data summary table presenting the raw data, including the number of data points and accessions, followed by the percentage retained after outlier correction. Heritability estimates are also provided for each dataset.

Dataset	Data points	Accessions	Data points kept	Accessions kept	Heritability
Agroscope spelt	3946	226	62.5%	94.7%	98.9%
Agroscope wheat	15286	810	72.2%	50.7%	97.5%
CREA wheat	5937	1353	80.6%	77.6%	88.3%
ICARDA wheat	57512	22606	47.8%	27.8%	65.7%
INIA wheat	1601	1260	30.7%	16.1%	89.3%
IPGR-Sadovo wheat	2491	504	98.5%	99.8%	96.6%
NARDI wheat	4267	750	42.2%	18.5%	72.4%
NPPC spring wheat	897	883	NA	NA	NA
NPPC winter wheat	5572	4850	16.4%	6.4%	75.2%
WUR wheat	794	678	15.0%	8.7%	75.0%

**Table 3:** **Plant height** data summary table presenting the raw data, including the number of data points and accessions, followed by the percentage retained after outlier correction. Heritability estimates are also provided for each dataset.

Dataset	Data points	Accessions	Data points kept	Accessions kept	Heritability
Agroscope spelt	1797	308	86.3%	55.2%	92.0%
Agroscope wheat	14039	810	74.3%	50.5%	99.5%
CREA wheat	5927	1353	91.3%	80.4%	94.7%
ICARDA wheat	54418	22267	83.7%	64.2%	67.6%
IHAR spring wheat	5376	2340	73.7%	54.6%	92.6%
IHAR winter wheat	10673	5267	81.9%	64.7%	93.2%
INIA wheat	1601	1260	34.0%	17.1%	66.0%
IPGR-Sadovo wheat	2489	504	98.6%	99.8%	96.7%
NARDI wheat	3706	748	59.1%	18.6%	92.2%
NPPC spring wheat	940	891	NA	NA	NA
NPPC winter wheat	6002	4885	29.6%	15.2%	93.6%
WUR wheat	3057	2231	48.2%	29.7%	69.4%

**Table 4:** **Thousand kernel weight** data summary table presenting the raw data, including the number of data points and accessions, followed by the percentage retained after outlier correction. Heritability estimates are also provided for each dataset.

Dataset	Data points	Accessions	Data points kept	Accessions kept	Heritability
Agroscope spelt	696	149	86.4%	67.1%	66.6%
Agroscope wheat	8791	201	82.8%	88.6%	97.0%
CREA wheat	1552	363	92.8%	75.5%	87.2%
ICARDA wheat	33366	15416	63.9%	35.4%	82.2%
IHAR spring wheat	5376	2340	76.5%	55.0%	82.2%
IHAR winter wheat	10673	5267	81.7%	64.8%	72.4%
INIA wheat	1512	1258	28.9%	15.8%	72.0%
IPGR-Sadovo wheat	2491	504	98.4%	100.0%	93.3%
NARDI wheat	1662	111	64.8%	59.5%	91.2%
NPPC spring wheat	564	535	NA	NA	NA
NPPC winter wheat	4387	3319	30.6%	16.8%	94.7%
WUR wheat	1063	1032	NA	NA	NA

Note on Fig. 5: the relatively high heritability estimated for TKW compared to days to heading and plant height in the ICARDA dataset likely reflects differences in the underlying data, an overestimation of replication due to the use of arithmetic means, and a possible overestimation of genotype variance.

## Usage Notes

**Fig. 6:**
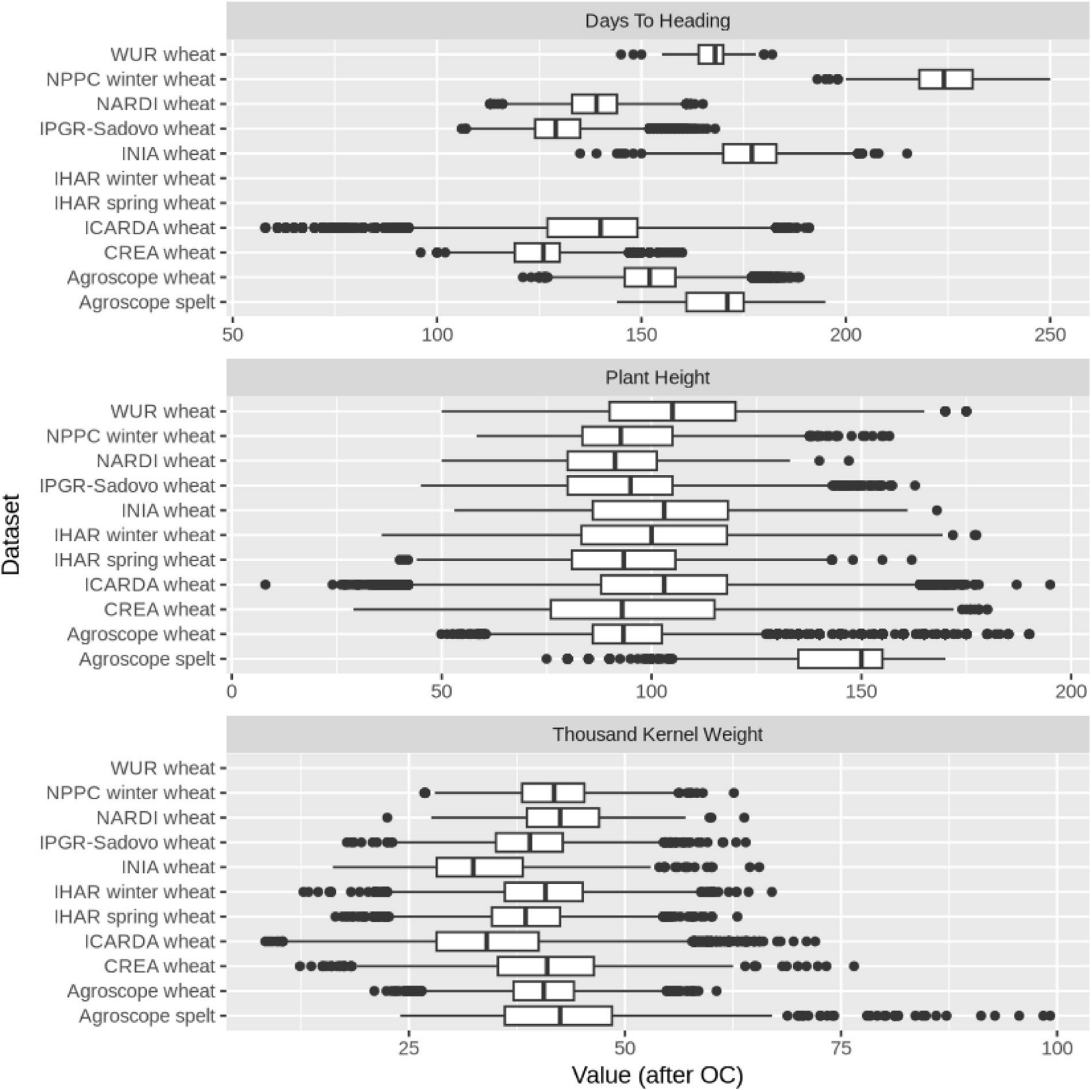
Distribution of the values for the 3 main traits studied across genebanks, after outlier correction. Cautionary note: phenotypic data is not directly comparable across genebanks, e.g. due to environmental variability as well as protocol differences (section “Phenotyping protocol”).

In this study, we provide comprehensive historical data on wheat genetic resources in accordance with the FAIR principles. The broad phenotypic diversity within the historical dataset of *Triticum* accessions is clearly evident when examining the wide distributions of the Best Linear Unbiased Estimates (BLUEs) for traits such as days to heading, plant height, and thousand kernel weight (Fig. 6, Supplementary Figure 1). These data are invaluable for researchers studying crop biodiversity and those seeking to expand genetic diversity for breeding purposes. Despite the non-orthogonal structure of the data, it serves also as a strong foundation for researchers in the field of biometrics. Enriching genebank documentation systems, which use persistent unique identifiers and harmonized passport data^12,13^, with interoperable phenotypic and genomic data, enables unambiguous identity verification and the detection of duplicates, enhancing germplasm curation^39^. Moreover, such data resources facilitate its integration with further historical and/or new datasets supporting informed selection for breeding and research. In summary, the presented data will be instrumental in transforming genebanks from passive repositories of crop biodiversity into dynamic bio-digital resource centres.

## Supplementary information

Supplementary Material

## Data Availability

The dataset is available both in a MIAPPE compliant tabular excel format at Recherche Data Gouv^29^ (10.57745/Y1VWIG, version 1.2), and as a FAIR Digital Object (FDO) in the Annotated Research Context (ARC) format at e!DAL-PGP^30^ (10.5447/IPK/2025/11, revision 2). Associated genotypic data was generated for a part of the observed germplasms. The raw sequence data is available at ENA^35^ (https://www.ebi.ac.uk/ena/browser/view/PRJEB49199, https://www.ebi.ac.uk/ena/browser/view/PRJEB81686). The variant data have been deposited in EVA^36^ (https://www.ebi.ac.uk/eva/?eva-study=PRJEB106343).

## Code availability

The code is available on GitHub (https://github.com/AGENTproject/historic_pheno_data_analysis/), Zenodo (10.5281/zenodo.15343013), and is archived in Software Heritage (https://archive.softwareheritage.org/swh:1:dir:a95895b0bfdbf346dbb31d656c9ef446c056caec). The code and artifacts from this repository are licensed under the permissive CC-BY-4.0, which allows any kind of reuse with the only condition of giving credit. The code is organised in one Jupyter R^40^ notebook per crop and per genebank. The computational environment is based on Conda packages. The main packages used are the *tidyverse*^41^ meta-package from conda-forge^42^, *bioconductor-multtest*^43^ from bioconda^44^, and the *asreml*^45^ proprietary R package. A full description of the computational environment is included in the repository, to enable reproducibility of the results.
