# Supplementary material for: Wheat historical phenotypic data from European genebanks as an important resource for research and breeding
Source: Sci Data. 2026 Mar 2;13:566. doi: 10.1038/s41597-026-06908-x (PMC13066454; doi:10.1038/s41597-026-06908-x)

Supplementary Material

Supplementary Tables

Supplementary Table 1: Triticum species proportions and their hosting genebanks.

Species	Genebank	Count
aestivum	IHAR	6947
	NPPC	3622
	WUR	1900
	CREA-CI	1329
	INIA	1260
	Agroscope	699
	NARDI	662
	IPGR-Sadovo	467
	ICARDA-Morocco	158
aethiopicum	ICARDA-Morocco	80
	IHAR	24
	NPPC	2
araraticum	Agroscope	1
	IHAR	1
boeoticum	Agroscope	3
	NPPC	2
	IHAR	1
carthlicum	NPPC	3
compactum	IHAR	19
	NPPC	4

dicoccoides	IHAR	5
	NPPC	2
dicoccon	NPPC	8
dicoccum	IHAR	41
	Agroscope	11
	NPPC	1
durocompac	Agroscope	1
durooblongum	Agroscope	1
durum	ICARDA-Morocco	364
	NPPC	136
	IHAR	125
	IPGR-Sadovo	36
	Agroscope	9
georcium	Agroscope	1
ispahanicum	Agroscope	2
	NPPC	1
karamyschevii	IHAR	3
	NPPC	2
kihare	IHAR	1
macha	IHAR	5
militinae	IHAR	1
	NPPC	1
monococcum	WUR	32
	NPPC	14
	IHAR	13
	Agroscope	1
persicum	IHAR	11

petropavlovskyi	NPPC	1
polonicum	IHAR	13
	NPPC	12
sp.	IHAR	279
	Agroscope	1
spelta	Agroscope	375
	IHAR	62
	NPPC	28
sphaerococcum	IHAR	4
	NPPC	3
timopheevii	IHAR	12
	WUR	8
	NPPC	3
turgidum	WUR	267
	IHAR	36
	NPPC	11
	CREA-CI	3
	Agroscope	1
vavilovii	IHAR	3
	NPPC	1
zhukovskyi	IHAR	1
NA	ICARDA-Morocco	22007
	NPPC	1935
	NARDI	88
	Agroscope	80
	WUR	32
	CREA-CI	21

	IPGR-Sadovo	1

Supplementary Table 2: Summary of environments in which accessions were tested.

Location	Phenotyping campaigns	Accessions tested	Data points
44.45, 26.52	40	750	19641
48.59, 17.82	38	5778	17977
47.43, 8.52	37	432	20619
52.21, 20.64	29	7259	124644
46.39, 6.23	28	804	4572
42.12, 24.93	28	504	24527
40.51, -3.31	22	1260	6331
46.91, 6.97	22	121	9702
47.62, 9.13	22	100	8776
46.38, 6.24	22	98	6936
46.67, 6.79	22	92	1633
46.65, 6.59	22	79	4496
46.77, 7.11	22	74	1040
52.31, 19.41	21	831	4124
46.33, 6.9	21	74	1231
47.42, 8.51	21	70	1565
35.93, 36.65	20	16402	61096
46.57, 23.78	20	16	1778
46.62, 6.58	19	80	3675
47.29, 7.72	18	65	1865

46.85, 7.19	15	68	984
51.99, 5.65	14	1195	3081
47.44, 8.68	14	72	1557
46.52, 6.64	14	20	632
45.65, 25.6	14	16	717
47, 7.45	13	46	668
47.39, 8.33	13	46	655
44.78, 24.85	13	6	393
46.45, 6.25	12	30	2552
47.06, 6.9	12	23	686
45.25, 9.42	10	1347	24127
47.04, 6.9	9	20	333
35.73, 37.18	7	1320	20841
46.89, 7.04	7	25	231
46.58, 6.54	6	15	69
33.6, -6.69	5	19347	47112
33.86, 35.99	5	357	1892
47.42, 8.54	5	32	137
46.55, 24.56	5	8	158
44.16, 28.46	5	6	157
33.91, 36	4	3198	12938
51.41, 4.14	4	450	518
47.32, 8.33	4	17	33
45.32, 8.37	3	405	873
51.7, 6.01	3	311	447
53.39, 6.31	3	258	258
45.3, 9.51	3	226	3704

42.13, 24.93	3	221	2871
46.81, 7.22	3	27	216
46.64, 6.64	3	19	904
47.05, 7.57	3	17	29
47.49, 7.93	3	17	29
47.67, 8.7	3	17	29
46.77, 7.25	3	14	43
46.96, 9.58	3	14	39
47.42, 8.69	3	9	27
47.87, 23.13	3	8	63
46.84, 6.97	3	7	139
46.15, 8.95	3	6	21
45.76, 21.23	3	5	109
47.71, 8.72	3	3	8
47.32, 9.18	3	2	24
47.45, 9.14	3	2	20
48.57, 17.74	2	177	385
52.49, 5.58	2	160	498
52.7, 5.7	2	157	161
51.98, 5.53	2	119	120
50.06, 19.94	2	26	168
47.63, 8.61	2	12	15
47.2, 8.07	2	8	12
45.92, 23.2	2	6	82
34, 36.2	1	222	1315
48.59, 17.81	1	195	381
32.85, 36.21	1	51	102

51.96, 5.65	1	28	55
41.65, 0.39	1	28	55
47.45, 8.74	1	22	22
46.91, 6.92	1	9	69
46.74, 6.6	1	8	38
46.41, 6.15	1	8	8
47.34, 8.54	1	7	7
47.18, 8.32	1	6	24
46.86, 26.85	1	5	55
46.47, 6.47	1	5	10
44.36, 23.82	1	4	41
47.38, 27.47	1	4	36
47.73, 26.68	1	4	35
47.07, 21.92	1	3	35
46.35, 25.8	1	3	27
45.97, 20.77	1	3	26
47.41, 8.08	1	3	3
45.27, 27.96	1	2	32
47.65, 26.26	1	2	21
46.38, 6.21	1	2	4
47.35, 8.11	1	2	2
47.41, 8.72	1	2	2
47.51, 8.01	1	1	1

Supplementary Figures

Supplementary Figure 1: Trait value distribution per Triticum species.

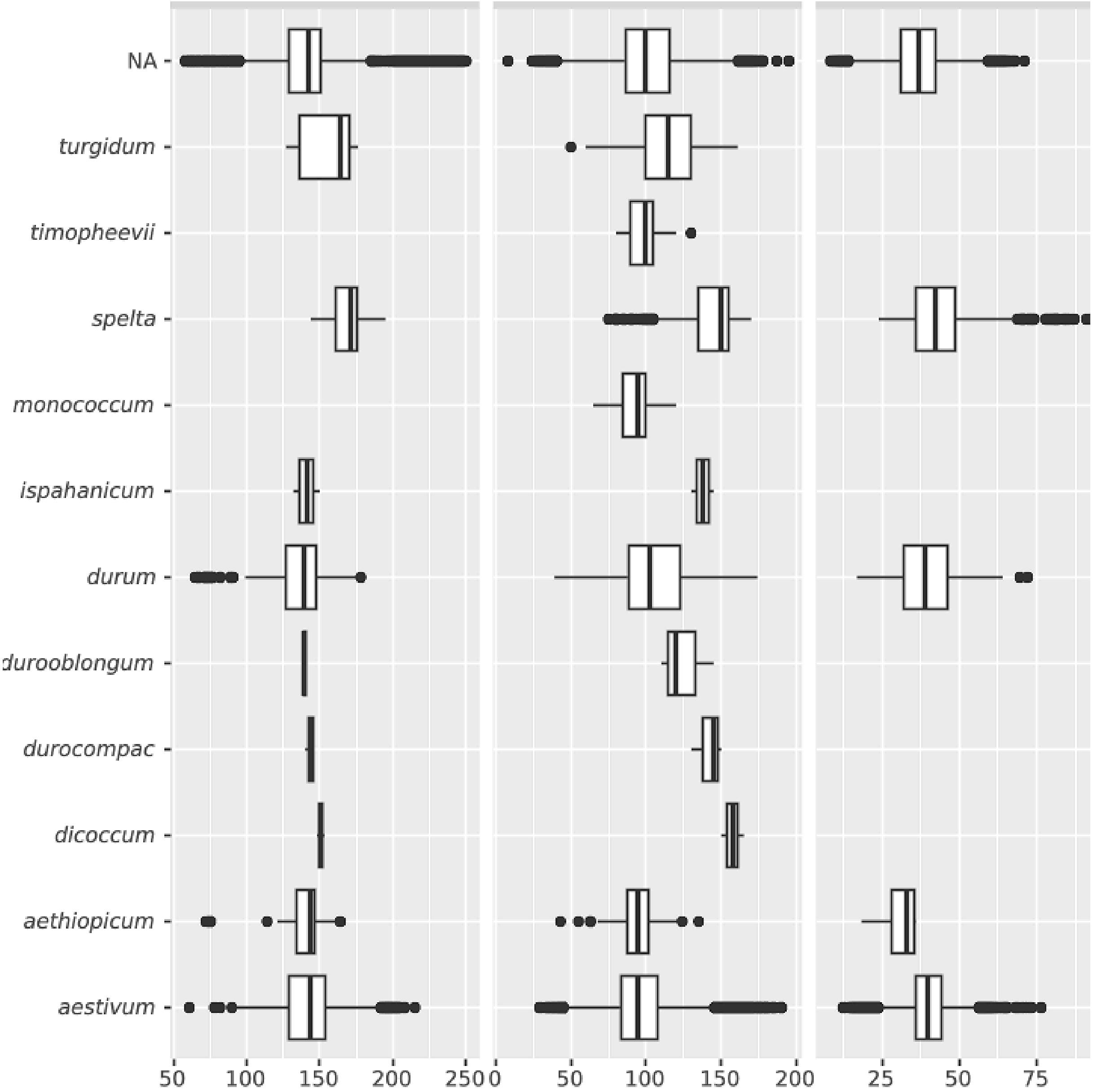

Supplement: Supplementary file 1 — Supplementary Material [file 41597_2026_6908_MOESM1_ESM.pdf]
